# Enabling Large-Scale IoT-Based Services through Elastic Publish/Subscribe

**DOI:** 10.3390/s17092148

**Published:** 2017-09-19

**Authors:** Sergio Vavassori, Javier Soriano, Rafael Fernández

**Affiliations:** School of Computer Science, Universidad Politécnica de Madrid, 28660-Boadilla del Monte, Madrid, Spain; jsoriano@fi.upm.es (J.S.); rfernandez@fi.upm.es (R.F.)

**Keywords:** content-based publish/subscribe, Chandy–Lamport algorithm, distributed snapshot, distributed system, Internet of Things

## Abstract

In this paper, we report an algorithm that is designed to leverage the cloud as infrastructure to support Internet of Things (IoT) by elastically scaling in/out so that IoT-based service users never stop receiving sensors’ data. This algorithm is able to provide an uninterrupted service to end users even during the scaling operation since its internal state repartitioning is transparent for publishers or subscribers; its scaling operation is time-bounded and depends only on the dimension of the state partitions to be transmitted to the different nodes. We describe its implementation in E-SilboPS, an elastic content-based publish/subscribe (CBPS) system specifically designed to support context-aware sensing and communication in IoT-based services. E-SilboPS is a key internal asset of the FIWARE IoT services enablement platform, which offers an architecture of components specifically designed to capture data from, or act upon, IoT devices as easily as reading/changing the value of attributes linked to context entities. In addition, we discuss the quantitative measurements used to evaluate the scale-out process, as well as the results of this evaluation. This new feature rounds out the context-aware content-based features of E-SilboPS by providing, for example, the necessary middleware for constructing dashboards and monitoring panels that are capable of dynamically changing queries and continuously handling data in IoT-based services.

## 1. Introduction

The Internet of Things (IoT) relies on a continuously growing number of interconnected uniquely addressable heterogeneous electronics (UAHE), including sensors, actuators, smart devices, embedded computers, etc., producing tremendous amount of data about the surrounding living environments to nourish an ever-growing number of services [1]. The large-scale nature of IoT-based services can be effectively and efficiently facilitated and supported via utilizing Cloud Computing infrastructures and platforms for providing flexible and extensive computational power, resource virtualization and high-capacity storage for data streams. Data brokerage [2] is a key concept for handling such a massive number of IoT-based services in a cloud environment. Since cloud environments are intrinsically dynamic, the same brokerage system has to handle variability, scale out to cope with new load, and scale in when the peak has passed in order to save resources. However, today’s cloud systems, like OpenStack [3] or OpenNebula [4], offer no more than a queuing system for delivering notifications from components or at best a topic-based publish/subscribe [5,6,7]. The same thing happens with publish/subscribe services available online like Amazon Web Services (AWS) Simple Notification Service [8], Google Cloud Pub/Sub [9] or Microsoft Azure Service Bus [10]. This is a limiting factor since it requires queue and topic management: publishers must decide in which queue or topic to publish their messages and interested components must decide from which queue they read. Content-based publish/subscribe systems (CBPS) [11,12] use dynamic message routing to avoid this limitation and are able to minimize message traffic through the distribution network.

Nonetheless, the classical network of brokers that made up the CBPS distribution network has some intrinsic scalability limits: subscriptions must be propagated to all brokers and the number of connections to the system is dependent on the number of brokers. Two different strategies have been developed so far in order to solve the first limitation: *covering* and *merging* subscriptions and using *advertisements* to avoid sending subscriptions if there are no matching publishers. *Covering* and *merging* are, however, computationally expensive and only provide a real gain for heavily colliding distributions. On the other hand, *advertisements* provide the specific and definite benefit of reducing subscription dissemination, even though computation will increase slightly in order to perform routing [11,13].

To overcome the second limitation, that is, the number of connections that the system can handle, the connection handling part needs to be decoupled from the routing part. Some systems like [14,15] or others that use a distributed hash table (DHT) architecture for matchers [16] provide this feature; however, most require complex coordination to provide an elastic service or simply rely on the hypervisor layer to perform live migration [17].

We used the CBPS model provided by SilboPS [18], which already provided a solution to efficiently share and use sensor data coming from ubiquitous Wireless Sensor Networks (WSN) across a plethora of applications, in order to implement an elastic version of it, called E-SilboPS. Support for dynamic state partitioning was required to properly deliver elasticity, and this meant that we had to restructure the internal architecture. Nonetheless, the Application Programming Interface (API) and message compatibility have been conserved to guarantee transparent substitution from the standard to the elastic version.

Our proposal allows for the provisioning of an elastic CBPS system capable of providing large-scale IoT environments with an uninterrupted service even during the scaling operation, which is a requirement for today’s large-scale IoT based Services involving billions of messages gathered from an ever-growing number of sensors.

The remainder of the paper is organized as follows: Section 2 shows the general architecture of the system and a brief review of the related work is presented in Section 3, highlighting the differences of our research with respect to other noteworthy systems; Section 4 describes the specific architecture of E-SilboPS, whereas Section 5 explains in details how the scaling algorithm works using a scale-out use case. Then, Section 6 analyzes the performances of E-SilboPS executing a scale-out with different configurations, detailing throughput, speedup and efficiency for each configuration in order to illustrate its strengths and the best deployment for a given load. Finally, we state our future goals and highlight the findings of this research in Section 7 and Section 8.

## 2. General Architecture

Connecting *objects* or *things* involves the need to overcome a set of problems arising in the different layers of the communication model. Using their data or acting upon them requires interaction with an ever-growing, heterogenous environment of devices running different protocols, due to the lack of globally accepted standards, dispersed and accessible through multiple wireless technologies. For this reason, approaching an IoT platform must enable intermediation, data gathering and data publishing functions.

**Figure 1:**
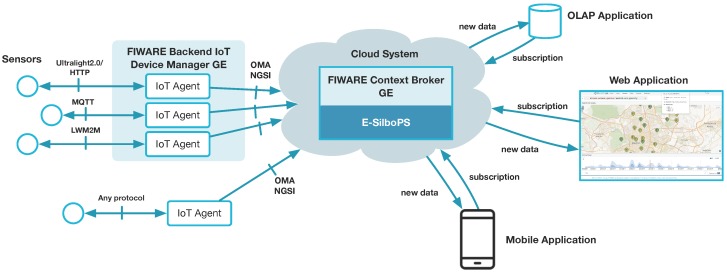
General architecture to support large scale IoT services. IoT sensors (**left**) are sending their data to the Context Broker and this one leverages E-SilboPS to relay messages to the interested subscribers (**right**).

FIWARE IoT services enablement platform [19] offers an architecture of components (called Generic Enablers or GEs) specifically designed to face these problems by allowing to capture data from, or act upon, IoT devices as easily as reading/changing the value of attributes linked to context entities. Figure 1 depicts the general architecture of this platform, which includes:

- The FIWARE backend IoT Device Management GE enables creation and configuration of IoT Agents (left part of Figure 1) that connect to sensor networks (ETSI M2M, MQTT, IETF CoAP, etc). IoT Agents solve the issues of heterogeneous environments where devices with different protocols approach to a common data format. IoT Agents act as mediators/translators between the protocols that devices use to send or receive information (HTTP Ultralight, MQTT, OMA Lightweight M2M), and a common language and data model across all the platform: FIWARE Next Generation Service Interfaces (NGSI) [20].
- The FIWARE Context Broker GE (center) to gather, publish, query and subscribe-to in-time IoT-enabled context information at large scale, in order to be used by hosted applications. The Context Broker implements the OMA NGSI-9/10 API [21] and it relays internally on the E-SilboPS component for all the publish/subscribe communication features. E-SilboPS provides the Context Broker with an elastic content-base publish/subscribe (CBPS) system specifically designed to support context-aware sensing and communication in IoT-based services.

FIWARE NGSI brings a simple yet powerful RESTful API enabling access to IoT-enabled context information. It is intended to manage the entire lifecycle of context information, including updates, queries, registrations, and subscriptions. Managing this information is possible since Context Broker keeps virtual representations of the physical devices. Interaction with devices will happen by updating and modifying the virtual representations attached/corresponding to them. From an architectural point of view, the Context Broker acts as a blackboard in a typical blackboard architecture. It is the core and control piece of the platform, in charge of interacting with the other components and agglutinate data.

By using this architecture, as shown in Figure 1, IoT devices will be represented in a FIWARE platform as NGSI entities in the Context Broker. This means that you can query or subscribe to changes of device parameters status by querying or subscribing to the corresponding NGSI entity attributes at the Context Broker. E-SilboPS represents a key internal platform asset to achieve elasticity in the distribution of context information mediated by the Context Broker in a cloud-enabled environment.

Subscribers can be whatever application or user is interested in the data and do not need to know where the actual sensor is or the specific format it uses to send its data, thanks to the common NGSI API. An example of those can be smartphones applications to allow mobile users to receive important updates, web application for a wider analysis or data-storage facility to perform On Line Analytical Processing (OLAP) or other data-intensive tasks (right part of) Figure 1.

**Figure 2:**
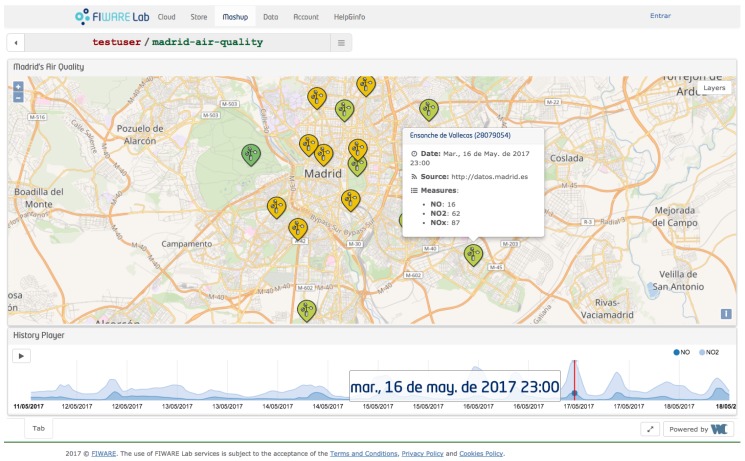
An example of subscriber application: the interactive map shows the various real-time contaminants measurements done by pollution station in the city of Madrid.

For the sake of clarity and for evaluation purposes, we use a running IoT example based on the air-pollution station deployed in the city of Madrid. In our example, we have gathered air pollution data generated from sensors mounted in fixed station (the left part of Figure 1), published to the Context Broker (center) and, by using specific subscriptions, consuming the new data (right) by displaying them in a web portal [22] to show real-time as well as historical time series categorized by pollutant, as shown in Figure 2.

## 3. Related Work

In the context of WSN, the adoption of decentralized publish-subscribe services is already established. These approaches are complementary to our work and can use our system as a way to connect various IoT services. For example, the work in [23] focuses on the internal WSN routing instead of the message distribution between IoT services, so it could benefit from using our solution to get connected to third party services.

Many elastic CBPS have been proposed over the last few years. However, most offer automatic scalability rather than proper elasticity. As a matter of fact, elasticity is the degree to which a system is able to adapt to workload changes by autonomously provisioning and deprovisioning resources to assure that the available resources *match* the current demand as closely as possible at any time [24]. This means that the application of the new configuration should not affect the offered service or require a *restart* of the system that always amounts to downtime.

The most noteworthy examples of systems are BlueDove, SEMAS and E-StreamHub [15,16,17]. BlueDove and SEMAS use a *fat Access Point* layer that handles the mechanism for selecting which matcher holds the subscriptions whose attributes are compatible with the current notifications, whereas matchers are organized in a P2P structure, typically using a DHT like Chord [25]. This approach is capable of selecting only the matchers that are of potential interest and it does not waste the time of others that already know that they have no subscriptions matching the notification. However, it requires some state duplication between the *Access Point* layer and the *Matcher* layer. Additionally, the fact that the access points include the state makes the scaling algorithm more complex since both layers have to be updated consistently.

On the other hand, E-StreamHub is the elastic version of Streamhub [14]. Streamhub has a cleaner architecture that is able to add and remove operators from each layer in a simpler manner. It also has stateless access points, and thus requires less complex algorithms to perform a consistent *change view*. In addition, the *Matcher* layer does not use a DHT, and it is a single hop from the *Access Point* layer, thereby reducing matching latency. Due to its implementation, however, it does no dynamic state partitioning; instead, it partitions into slices its internal state during startup and then moves the slices from one Virtual Machine (VM) to another in order to scale out.

We argue that this approach does not provide proper *elasticity* since it limits a priori the maximum achievable scale-out on the grounds not of zero efficiency [26] or a slowdown of the speedup but simply because the partitioning parameter is wrongly forecast during startup. Irrespective of the chosen partitioning parameter value, the system will have an intrinsic avoidable overhead for all less-demanding workload, as well as a *fixed* upper bound for scalability. This goes completely against the definition given by [24].

As a matter of fact, this strategy can be seen as an application-specific version of VM live migration already offered by today’s hypervisors like KVM, Xen, VirtualBox or VMWare. Application-agnostic hypervisor-based VM live migration is more valuable in the general case, but it has the same limitation as E-StreamHub, specifically with respect to dynamic state partitioning. In Section 4, we show how E-SilboPS can overcome the above weakness, while retaining the cleaner and simpler architecture of E-StreamHub.

## 4. Concept

The system should have a global elasticity property in order to overcome the weakness of sizing a scalable monitoring system with an a priori deployment, namely wasting resources or underperforming monitoring because of the changing load. In our approach, we have added this property to all operators. In this way, we can adapt each layer to the specific environment without worrying about providing a setup that fits from the start. In addition, temporary deviations from the standard usage pattern, like load spikes, can be handled without having to restart the system.

**Figure 3:**
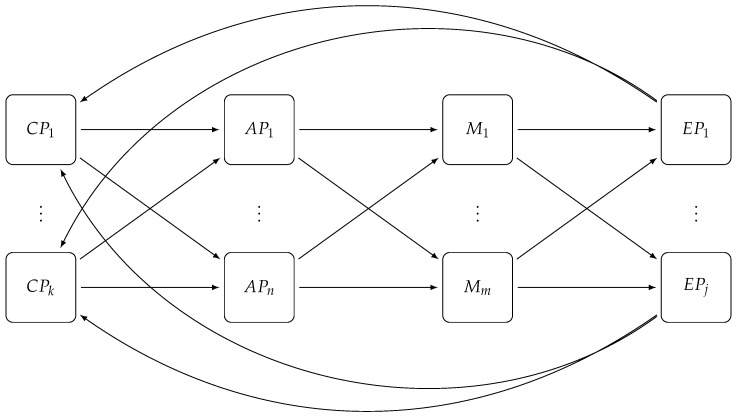
E-SilboPS architecture for the all layers: *Connection Point*, *Access Point*, *Matcher* and *Exit Point*. Notifications and subscriptions are coming from the *Connection Point* layer to the *Access Point* layer in order to be dispatched to the correct *Matcher* layer instance ($M_{1}$⋯$M_{n}$) and then it will be sent to a specific *Exit Point* instance to collect all responses and avoid message duplication. Finally, the message will be sent to the selected *Connection Point* to be delivered to clients. In this figure, the Distributed Coordinator, since it has a direct connection to each slice of each layer, has been omitted in order to keep the diagram clean.

E-SilboPS is divided into four layers, as shown in Figure 3, similar to the engine described in [14,17], and each operator layer can scale in and out independently of the others. Some orchestration between layers is needed, however, to maintain state consistency, and it is done by creating and removing nodes from the Distributed Coordinator (DC) in order to fire events to the interested layers; the DC is implemented using Zookeeper (We use ZooKeeper to ensure a reliable distributed coordination between operator slices. [27]) These four layers are:

**Access Point (AP)**  dispatches incoming subscriptions to the correct *matcher* and broadcasts notifications to all matchers.
**Matchers (M)**  store incoming subscriptions in the internal structure, match notifications against subscriptions, and dispatch the local result set to the *Exit Point* layer.
**Exit Point (EP)**  collects all partial results sets coming from the *Matcher* layer, which it merges in the final results set and then sends the notification to all *Connection Points* found in this set.
**Connection Point (CP)**  represents the system entry and exit point; it handles the connection with clients and sends the received notifications to the interested subscribers; it forwards subscriber subscriptions and publisher notifications to the *Access Point*.

To perform proper scaling [24,28], the system must be able to match the variable demand of workload and, at the same time, control the cost of the deployed infrastructure. Our solution uses the *Chandy–Lamport algorithm* [29], also known as the *snapshot algorithm*, to create a consistent cut of the global state of the system. It then executes the algorithm that we designed to transfer the state throughout the system, repartitioning it according to the new available slices. One noteworthy feature of our algorithm is that it is capable of matching notifications while restructuring its internal state and has a very short no-matching time before switching to the new state.

## 5. The Algorithm

In this section, we explain our scaling algorithm: there are three possible layer reconfigurations, one for each operator except for *Connection Point*. Coordination with other layers may be required depending on the layer and operator state. For each kind of reconfiguration, we will list the operations to be done by each layer in order to keep a consistent state.

### 5.1. Access Point Scaling

Access point scaling is straightforward since it is a stateless operator, and no coordination is required because it is usually bound with a *Connection Point*. This means that layer reconfiguration is limited to adding or removing target operators and opening or closing connections to the *Matcher* layer.

**Access Point layer:**  start or stop instances according to the new configuration.
**Matcher layer:**  no action.
**Exit Point layer:**  no action.

### 5.2. Matcher Scaling

**Figure 4:**
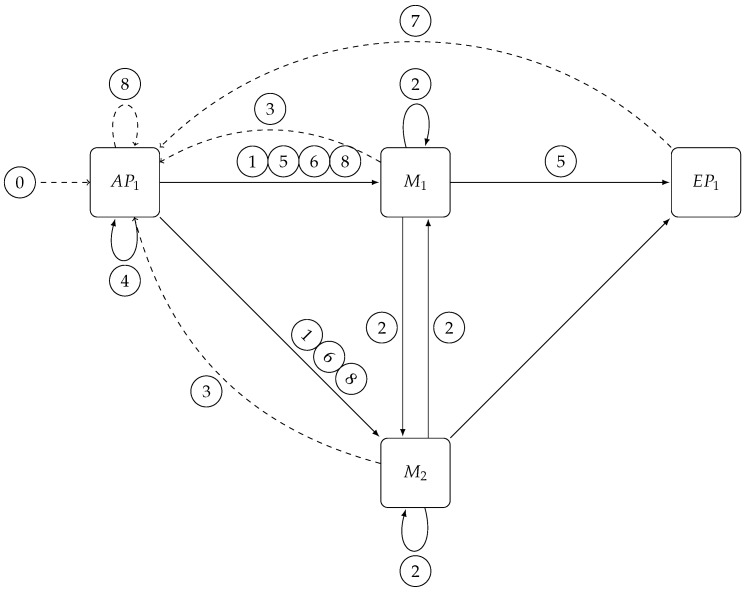
Messages exchanged during *Matcher* layer scale-out: solid lines represent connections between operators used by the system to send notifications and subscriptions. Dashed lines represent Distributed Coordinator-mediated messages used to synchronize layers, typically to unlock all operators from a layer waiting for another layer to complete its task.

As shown in Figure 4, the scaling of the *Matcher* layer requires coordination with the other two layers since *Access Point* will need to use a new selector and buffer subscriptions and notifications, whereas *Exit Point* will have to change their internal state to correctly collect notifications coming from the new *Matcher* configuration.

The scaling process starts by sending a Start Scale-In/Out message to all Access Points ⓪; upon receiving this message each access point starts buffering subscriptions and forwards the same message, together with the new *selector* that they have to use, to all existing matchers ①. This renders the state of the *Matcher* layer temporarily unchangeable, although, at the same time, it is still able to process notifications. Thus, this phase is transparent for *publishers*, and *subscribers* will still receive notifications as usual, although they will not see any updates of the subscriptions that have changed in the meantime.

As soon as a matcher receives the first Start Scale-In/Out message, it connects to all matchers of the new configuration and partitions its internal state using the *selector* included in the received message. It then sends this new state to the respective matchers, itself included ②. In this way, all matchers know in advance from which matcher they will receive the new state, that is, from the matchers present in the *new* configuration in the event of a *scale-out* or from the matchers in the *old* configuration in the event of a *scale-in*.

When a matcher has received all state information, it sends a Change-Ready message to the Distributed Coordinator to notify the *Access Point* layer ③. This layer will be unlocked with a Change-Matcher-Ready when all matchers have finished ④. The Change-Matcher-Ready message signals to the *Access Point* layer to enqueue notifications and to send a Change-Matcher-On-Exit Point message to all old matchers ⑤ and a Change-Matcher to all (old+new) matchers ⑥ so that they can swap their internal state for the new one.

The Change-Matcher-On-Exit Point message must precede Change-Matcher because it is forwarded upon arrival at the *Matcher* layer to the *Exit Point* layer. This creates a consistent change of view of the *Matcher* layer state from the *Exit Point* layer perspective without direct synchronization between matchers. This message must be sent to all matchers in order to prevent subsequent notifications reaching a newly added matcher and being processed faster than the last notification sent before the state change. Otherwise, the exit point would associate this new notification with the wrong number of matchers, which, in the event of *scale-out*, would lead to the received subset of subscribers being notified too early. This would leave in starvation the remaining set of subscribers at the same exit point. In the event of *scale-in*, the notification will never be delivered to subscribers because the exit point will not receive all the expected sets for this purpose.

When an exit point receives the Change-Matcher-On-Exit Point message, it reads the current number of matchers from Distributed Coordinator and notifies its new state. The number read from the configuration will be immediately used as the new threshold before pushing notifications to subscribers.

Once all exit points have been updated, the Distributed Coordinator will send an Exit Point-Matcher-Updated to *Access Point* layer ⑦ to flush buffered notifications if *scaling-out* and wait for all access points to finish the operation. It will then send a Scale-End message to the *Access Point* layer ⑧, which will be forwarded to the *Matcher* layer and will close all unnecessary connections. On receiving the Scale-End message, each matcher will close its connections to others but will not forward this message to the *Exit Point* layer since no operation is needed for this purpose.

**Access Point layer** 
  - When receiving a Start Scale-In/Out message with a new matcher selector:
    1. Continue to broadcast notifications to all current matchers
    2. Buffer subscriptions in a queue
    3. Send Start Scale-In/Out message to all (old+new) matchers with the new matcher selector to be used
      
  - When receiving a Change-Ready message:
    1. If in the *scale-out* phase, the notifications are no longer sent. (The notifications sent after *change view* could enter the new matcher instance, being matched sooner than other notifications from other matchers and reaching the Exit Point before the last notification of the previous group.)
    2. The Change-Matcher-On-Exit Point message is sent to all old matchers
    3. The Change-Matcher-Ready is sent to all (old+new) matchers
    4. The current selector is exchanged for the new selector received from *Access Point*
    5. While waiting Exit Point Matcher-Updated sends enqueued subscriptions to matchers
      
  - When receiving a Exit Point Matcher-Updated message:
    1. If it is in *scale-out* phase, flush enqueued notifications
    2. Resume sending notifications
    3. Signal Scale-End to the Distributed Coordinator
    
**Matcher layer** 
  - When the Matcher has received a Start Scale-In/Out message with a new matcher selector from all *Access Points*:
    1. Notifications continue to pass through the matcher using current matching algorithm (No subscriptions can arrive because they are buffered at Access Point layer.)
    2. Snapshot matcher’s *Subscription Map*
    3. Map snapshotted subscriptions set with new matcher selector and store the result in Map<Slice, Map<ConnectionID, Set<Subscription>>>
    4. Send the new mapping to each matcher and to itself
      
  - When receiving a Add-Matcher-State message:
    1. The subscription set is added to the new matcher state
    2. When all Add-Matcher-State messages have been received, the Change-Ready message is sent to *Access Point* through the Distributed Coordinator
      
  - When receiving Change-Matcher-On-Exit Point:
    1. Forward it to all Exit Points
      
  - When receiving a Change-Matcher-Ready message:
    1. Swap matcher state for new one
    2. Start processing notifications and subscriptions
    
**Exit Point layer** 
  - When receiving a Change-Matcher-On-Exit Point message:
    1. The new number of matchers is read from the DC and used as the current limit for notifications
    2. Exit Point-Changed is sent to DC

### 5.3. Exit Point Scaling

**Figure 5:**
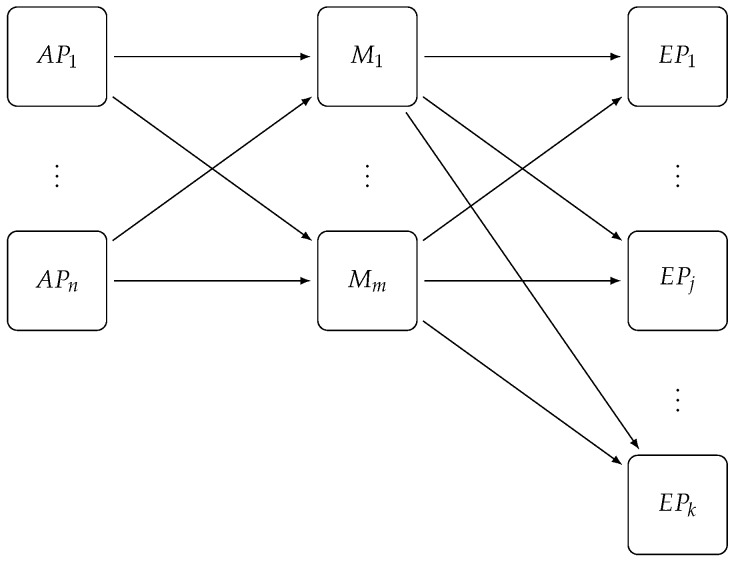
*Exit Point* layer scale-out: exit points from j+1 to *k* are added to the current configuration.

*Exit Point* layer scaling (see Figure 5) has to be coordinated with the *Matcher* layer because a consistent cut of notification must be done. Even though no direct mechanism of synchronization between matchers is required, each matcher has to perform a set of operations such that the added (or removed) exit point instances will be used only after a consistent *change view*.

An Exit-Point-Scaling message is first sent to access points which they then broadcast to matchers. The matchers use this message to create the new exit point *selector* and buffer notifications on an access point basis. Once it has received the respective message from all access points, the matcher processes all buffered notifications using the new exit point *selector* and sends a message to the Distributed Coordinator to notify its reconfiguration. After collecting all reconfiguration messages from matchers, the Distributed Coordinator sends a Scale-End message to the *Access Point* layer to clean up connections in the same manner as for *Matcher* layer scaling.

**Figure 6:**
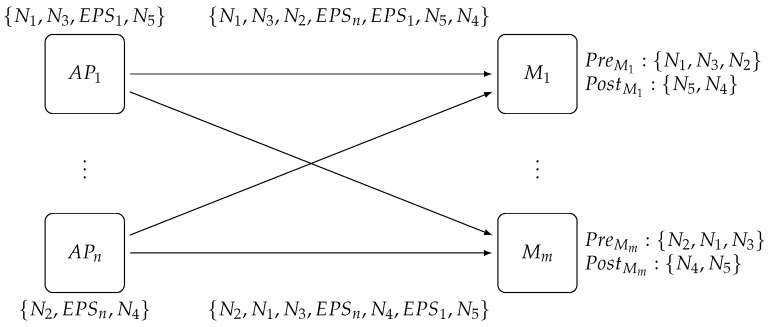
*Matcher* layer notifications cut: $AP_{1}$ sends odd notifications ($N_{1}\cdots N_{5}$), whereas $AP_{n}$ sends even notifications ($N_{2}\cdots N_{4}$). $EPS_{1}$ and $EPS_{n}$ represent the Exit Point-Scaling messages broadcast by each access point to all matchers.

As shown in Figure 6, $AP_{1}$ sends notifications $N_{1}$, $N_{3}$ and $N_{5}$, whereas $AP_{2}$ sends notifications $N_{2}$ and $N_{4}$. However, due to the independent FIFO channels that connect access points to matchers, the order of delivery viewed by $M_{1}$ is $N_{1}$, $N_{3}$ and $N_{2}$, as opposed to $N_{2}$, $N_{1}$ and $N_{3}$, as viewed by $M_{m}$, before reception of the two Exit Point-Scaling messages. In addition, $M_{m}$ receives $N_{4}$ before $EPS_{1}$, but it knows that this notification will be in the Post set because it has already received $EPS_{n}$ from the same channel.

This proves that, even if the order of the messages received by matchers differs, their Pre and Post sets contain the same elements, so they can process notifications according to a *consistent cut*. In addition, the relative ordering of notifications coming from each access point is preserved, so, from a *publisher*’s point of view, no out-of-order matching is performed.

**Access Point layer** 
  - When receiving Exit Point-Scaling:
    1. Forward Exit Point-Scaling to all matchers
    
**Matcher layer** 
  - When receiving first Exit Point-Scaling:
    1. Read from the DC the number of *Access Point* and *Exit Point* instances
    2. Create the new *Exit Point* selector
    3. Start enqueueing notifications from that *Access Point* slice
      
  - When receiving subsequent Exit Point-Scaling:
    1. Notifications from that *Access Point* slice are enqueued
      
  - When receiving last Exit Point-Scaling:
    1. The new *Exit Point* selector is adopted and all enqueued notifications are flushed
    2. DONE message is sent to the DC to signal the end of current matcher reconfiguration
**Exit Point layer** 
  - Instances are added or removed according to the new configuration

## 6. Evaluation

To evaluate how our architecture will operate and deliver messages coming from IoT sensors to all the interested subscribers, we implemented E-SilboPS.

E-SilboPS is the elastic version derived from SilboPS [30], our Java implementation of SIENA [13,18] with improved context management. As usual for P/S systems, our software does not support transactional messages, but offers FIFO channels with reliable delivery. This implies that no message loss will be observed, unless there is a link failure, of which both channel endpoints are aware. Messages are processed in the same order as they arrive at the operator instances irrespective of their sending time. The software is available for research purpose upon request.

We ran our experiment on an Intel i7 860 @ 2.80 GHz machine equipped with 12 GiB of RAM running Linux 4.2 and OpenJDK 7u91.

Unfortunately, we cannot compare directly E-SilboPS against E-StreamHub for two reasons: first, in their article, they wrote only the matching fraction of subscription per each notification, but there is no information about the distribution of the attributes to create those notifications and subscriptions as well as their value or their cardinality, so it is impossible to re-create the same environment. Secondly, we were not able to find where to download E-StreamHub and thus we could not run it within our environment. Nonetheless, since our aim is not to show that our system is faster that E-StreamHub in some environment but that state repartitioning can be achieved with reasonable performance, we do not think the lack of a direct comparison is a blocking point.

At first, we tested throughput performance with different configuration deployments to find out where the processing time was dominated by matching time instead of network time. We started our tests with a 1-1-1 configuration, that is, one access point, one matcher and one exit point with the addition of one connection point bound to the access point. Before measuring performance, we created 100 k notifications and one million subscriptions in order to assure that object creation time did not affect the measured throughput. In addition, the notifications were created to match at least one subscription in order to be able to constantly measure notification throughput.

**Figure 7:**
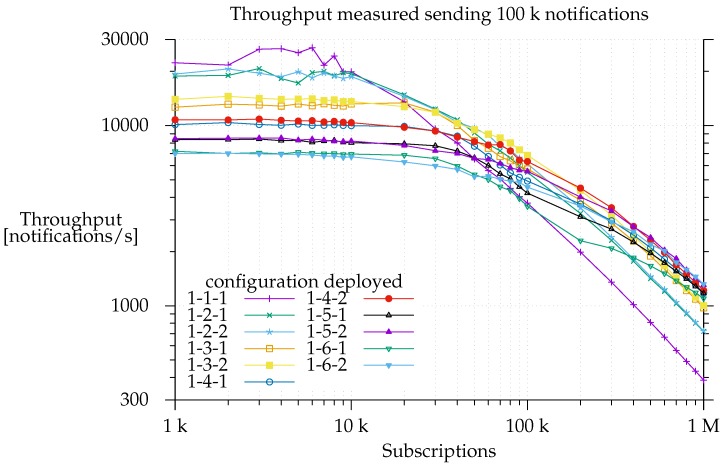
Notification throughput with different configurations: the triplet in the legend represents the number of instances deployed for *Access Point*, *Matcher* and *Exit Point*.

This is a worst-case scenario setup since the system has to deliver all the notifications, whereas, in a real environment, some notifications will not be delivered because no matching subscriptions will have been sent. As shown in Figure 7, when E-SilboPS was deployed with a 1-1-1 configuration, notification throughput started to slow down just after the system was loaded with 10 k subscriptions. It descended at a constant rate, and processing time started to be dominated by matching as of 20 k subscriptions. This means that the constant throughput on the left side of the graph is due to network saturation. This limits the amount of messages that an operator can process [31].

This is proven by the fact that there is a decrease in notification throughput when the number of operators, and consequently the number of messages passing to the network layer, increases, but the workload is unchanged. Unsurprisingly, minimum notification throughput is observed with the configuration deployment that has the maximum number of elements (1-6-2). In the same way, we find that increasing the numbers of matchers on the right side of the graph, where the matching time is dominant, improves throughput, because, on a per operator basis, the state is smaller, and thus it takes less time to calculate the partial set of subscribers. This is really important since it proves that the current architecture is able to scale out, which is a prerequisite for elasticity [24].

Another interesting point is that the addition of more matchers not only increases the throughput but also changes the slope of the curve. This is consistent with the region in the center of the graph where each matcher is working. If there are fewer subscriptions, the processing time is divided more equally between network time and matching time, and, ideally, this division will be such that the two are balanced leading to 100% usage of both resources [26,31].

**Figure 8:**
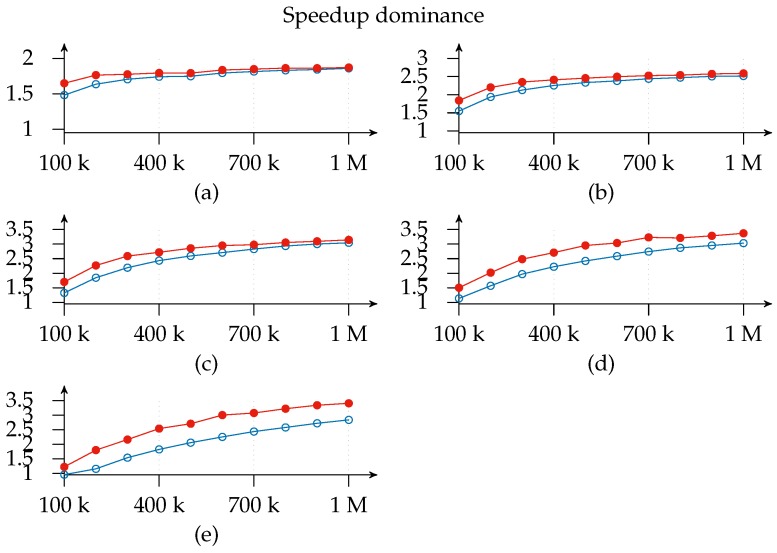
Notification throughput speedup dominance of 1-*x*-2 over 1-*x*-1: the line with filled circles plots the speedup for the 1-*x*-2 configuration, whereas the line with empty circles plots the speedup for the 1-*x*-1 configuration; plots (**a**–**e**) represent matcher instances, namely *x*, scaling from 2 to 6.

Note also that it is in the region between 50 k and 300 k subscriptions where performance differences between configurations that have only one exit point and others that have two start to appear. As a matter of fact, the 1-*x*-2 configuration always outperforms the 1-*x*-1 configuration, and the difference is greater the more matchers it has, as shown in Figure 8.

This means that the *Exit Point* layer does a good job of offloading work from matchers in this region, namely by reducing the partial sets of subscribers to a single set. On the contrary, network saturation limits the total throughput on the left of the graph, hiding any potential gain, whereas the matchers are the factor limiting notification throughput on the right.

**Figure 9:**
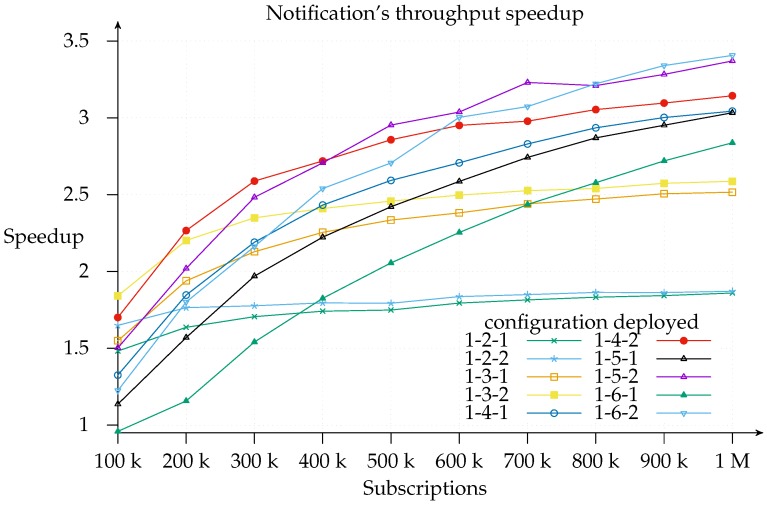
Notification throughput speedup for different configurations with an increasing subscriptions load.

**Figure 10:**
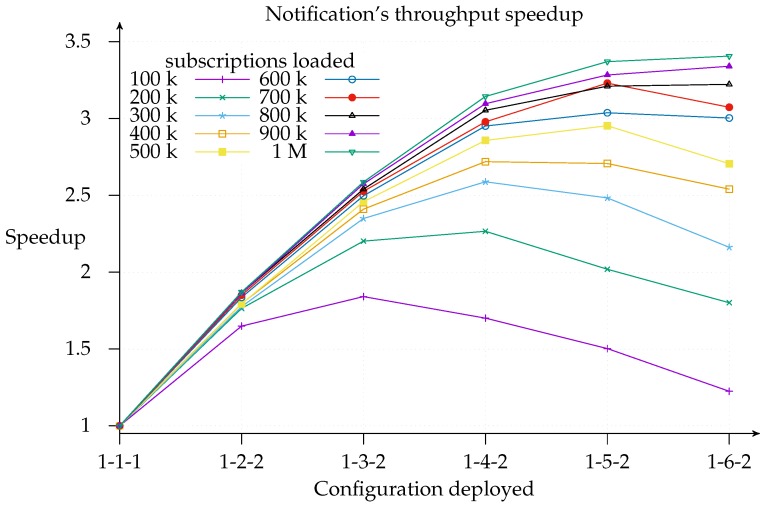
Notification throughput speedup sliced on 1-*x*-2 configurations.

**Figure 11:**
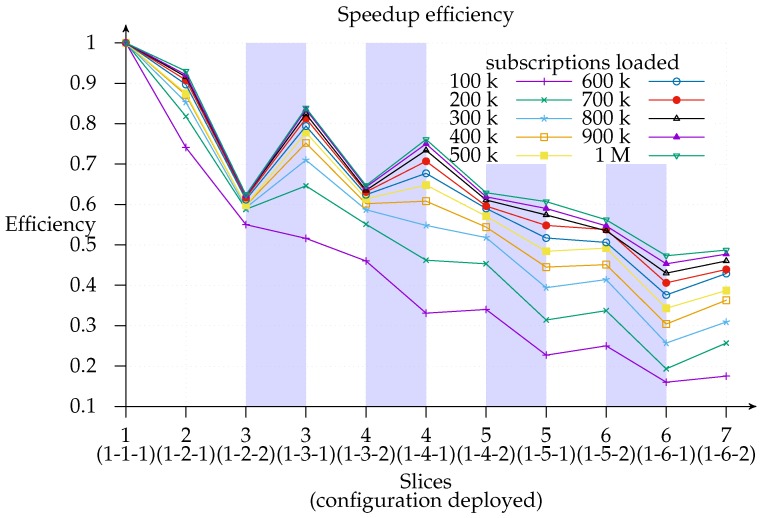
Notification throughput speedup efficiency: a horizontal line means equal efficiency, the highlighted region represents an equal number of slices where a horizontal line also denotes an equal speedup, whereas equal speedup is represented by the local hyperbola in the white region.

Finally, note for Figure 7 that, on the far right, the speedup gained by adding more and more matchers is downward, which is sub-linear. This applies to total efficiency, as shown in Figure 9, Figure 10 and Figure 11 too. This is consistent with Amdahl’s law, Gustafson’s law [32,33] and Eager [26]. Even so, the notification throughput provided using the 1-6-2 was 3.4 times greater than using the 1-1-1 configuration.

Figure 10 is useful for cost-benefit analysis of the scaling, since it shows where, for a given configuration deployment and load, it is worth adding another matcher. We find that the top speedup for a 100 k load is achieved with a 1-3-2 configuration. This means that adding more resources simply results in a worse throughput because of message overhead. Similarly, a workload increase pushes the curve’s peak to the right of the graph.

For clarity’s sake, Figure 10 shows the speedup for 1-*x*-2 deployments only, since, as shown in Figure 8 above, the 1-*x*-2 configuration always outperforms 1-*x*-1. However, the same does not apply with respect to efficiency, which is computed as a ratio of speedup to the number of instances (processors) used, that is, $E\left(n\right)=\frac{S(n)}{n}$ [26]. Figure 11 shows how efficiency decreases as the number of slices increases and the 1-*x*-1 configuration is more efficient than the 1-*x*-2 configuration in most cases and especially with deployments with not many instances. Additionally, this graph indicates the layer to which it is better to add a new slice since deployments with the same number of slices (highlighted regions) can be compared and a cost-benefit analysis of adding a second exit point (white regions) can be conducted. As a matter of fact, we find that the most efficient deployment for the three-slice region for all loads except 100 k is the configuration with only one exit point. This means that matcher requirements are greater than exit point utility. The same applies for the four-slice region, although the threshold is raised to a 400 k subscriptions load. This is consistent with expectations since the speedup *contribution margin* of each new added matcher will be smaller than its predecessor’s. This could be modeled in an *open* cloud using the Shapley value [34,35].

Likewise, we observe that adding an exit point in the five- and six-matcher region will help to improve both efficiency and speedup: this suggests that it is better to add an exit point than a matcher in such deployments. Thus, we can infer, as a rule of thumb, that one exit point is necessary for every four matcher instances, irrespective of the number of loaded subscriptions.

**Figure 12:**
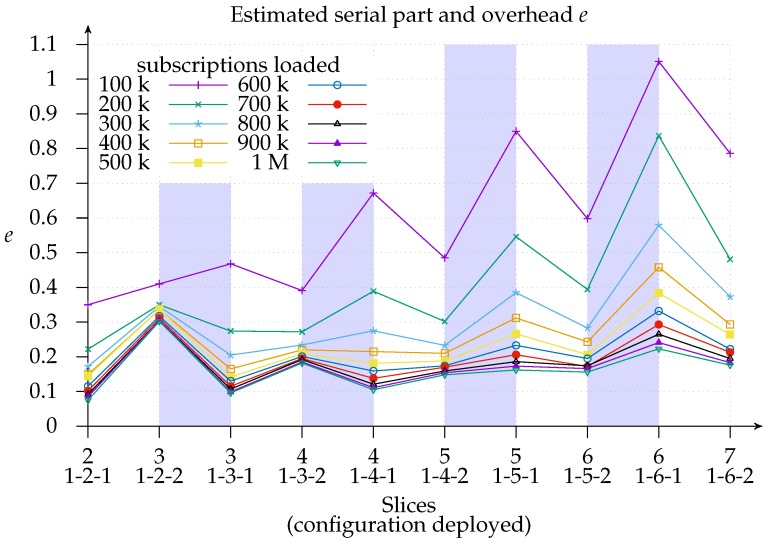
Estimation *e* of the serial part and overhead using the Karp–Flatt metric: the smallest load (100 k) clearly yields a larger overhead, whereas the biggest load (1 M) reduces overhead to 10%. This reduction is present in all configurations deployed and is directly proportional to the number of matchers. However, the number of exit points has a positive effect in that it lowers *e*. Therefore, the estimation of *e* can be attributed to *network* overhead rather than to the *intrinsic* serial part of matching notifications.

Finally, Figure 12 shows the estimated Karp–Flatt metric $e=\frac{\frac{1}{s}-\frac{1}{p}}{1-\frac{1}{p}}$ of configuration deployments, where *s* represents the speedup and *p* the number of slices (processors) used. This metric is used to estimate the serial part and the overhead from experimental data [36]. It is, therefore, a valuable metric for use in conjunction with efficiency, since it gives an insight into whether the decreased efficiency is due to the limits of parallelism or overhead. As we can see, we are experiencing *network* overhead with a 100 k load since adding more matchers increases the estimated *e*, especially with the 1-*x*-1 configuration. Looking at how the same deployment behaves with a larger subscriptions load, we find proof that the overhead is network related: the estimated *e* sinks to the 10–20% region.

In order to measure system behavior during scale-out, we loaded the system with 100 k subscriptions and set up a constant stream of 2000 notifications/s and 1000 subscriptions/s; these are the orders of magnitude normally used by publish subscribe systems like SIENA, PADRES and others to evaluate how the prototype performs under load, they are related to the machine used. Thus, we can examine the throughput of notifications delivered to the subscriber to see how the different *Matcher* layer scale-out phases affect the throughput of notifications. We ran this test with all deployments, finding that all the results were very similar.

**Figure 13:**
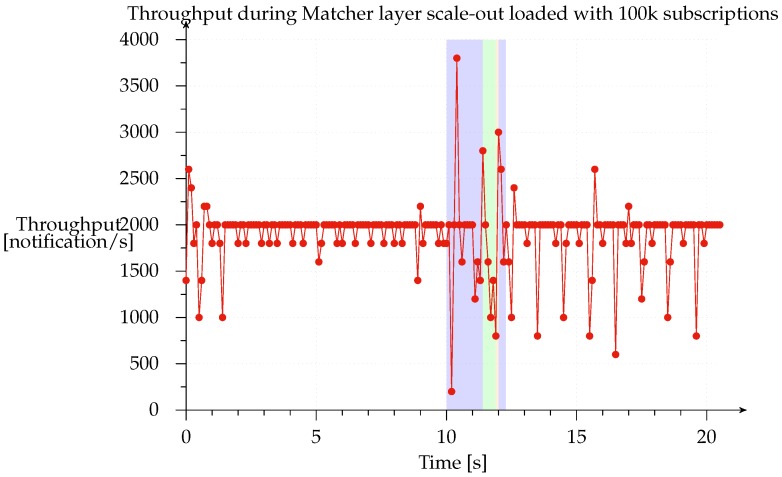
Notification throughput with subscriptions during scale-out of *Matcher* layer: the deployed system configuration was 1-1-1, scaling out to 1-3-1 and pre-loaded with 100 k subscriptions. The red line is the notification throughput delivered to subscribers while publishing 2000 notifications/s and adding 1000 subscriptions/s.

Figure 13 shows the scale-out from the 1-1-1 to the 1-3-1 configuration. The red line represents notification throughput received from the subscriber and the highlighted blue region represents the time during which the system was scaling out. Clearly, the system continues to process notifications while the subscriptions are buffered in the access points and matchers are switching to their new state, after which the access point starts buffering notifications and flushes all its buffered subscriptions (green region). This phase is illustrated in the graph from seconds 11.4 to 11.9 (490 ms). The matchers then have their new state updated so that they can reprocess the notifications. During the last phase (narrow orange band), which takes place from seconds 11.9 to 12.0 (98 ms), all notifications, buffered while sending the previously buffered subscriptions, are flushed. Obviously, since each matcher operates independently of the others, throughput is highly variable. The normal throughput of 2000 notifications/s is restored at the end of the scale-out, meaning that all internal buffers have been emptied.

We found that notification and subscription throughput has a linear impact on the duration of the scaling window. However, the system always completes the scale-out in a finite amount of time since the size of the buffers that it has to empty is determined by the time taken to complete each phase and the buffers are not updated with new subscriptions or notifications once a phase is complete. In the test that we performed, the time taken to scale out the system ranged from 1 to 8 s with notification and subscription throughput ranging from 1000 to 8000 messages per second.

This result shows how dynamic state partitioning can be achieved in an acceptable amount of time, thereby solving the problem of forecasting the maximum number of slices required during peak loads and providing the system with the most efficient configuration for the actual load. This is the major contribution of our research on implementing a proper *elastic* content-based publish/subscribe system. It differs from other systems [17] that use pre-state partitioning and simply move slices around. These systems offer no more than application-specific optimized versions of the VM migration provided by all major hypervisors today.

## 7. Future Work

E-SilboPS currently depends on an external system to decide when it has to scale in or out. This system has to make this decision based on current resource usage, as well as delivered performance. The use of metrics that measure only CPU or memory usage is not very suitable for a cloud system because they are only helpful for the technicians that manage the cloud. Users neither know nor care about how much CPU or how many machines are used, they are concerned about service quality such as Service Level Agreement (SLA), their user experience and billing cost. This means that the software that decides when to scale E-SilboPS in or out should have access to the above metrics and should possibly predict peaks of usage with respect to the SLA. At the same time, it should have knowledge of the performance efficiency and monetary cost of the current configuration, which should be optimum [37,38,39,40,41,42].

With E-SilboPS, we were able to add elasticity to a content-based publish/subscribe system. One possible improvement of our current model is to offer slice *replacement*. As a matter of fact, E-SilboPS manages slices of the same operator as a stack. This means that, in order to remove a specific machine, the system needs should scale in until that slice is no longer used. On the other hand, a better approach for this kind of operation is to use the VM migration offered by today’s hypervisors: thus, the VM containing the slice can be moved without any system scaling.

A second improvement is to take full advantage of the availability of network multicast: this will reduce the number of messages to be sent and CPU usage. This is warranted thorough validation since it could perform as well as the current implementation in a virtualized environment, where, unlike high-end routers, there are no physical processors dedicated to each endpoint.

Another potentially interesting feature deserving support is multilayer scalability: the capacity to scale all three layers in and out independently of each other within a single scaling transaction. However, this should be weighted against the increased complexity of the algorithm. Apart from in-field validation of the use case, we also need to check that it delivers the expected improvements or instead merely increases software complexity.

The last feature that we think would add value to the system is the capability to handover connections between connection points without disconnecting clients: this feature would enable load balancing by repartitioning connections looking at producer or consumer throughput.

Future work will be carried out within a number of use cases of the IoF2020 Horizon 2020 project [43], such as the Poultry Chain Management [44] one, in which we will deploy our proposed architecture in a distributed environment to collect/monitor a huge amount of data coming from three critical points in the poultry chain (i.e., at the farm, during transport, and in the slaughter house), to later provide daily insights and early warnings based on the available data. This scenario will allow us to test how E-SilboPS performs in a real large-scale IoT scenario, handling big data, and being connected through different networks with varying speed and latency.

## 8. Conclusions

In this article, we have presented a proper *elastic* content-based publish/subscribe system that supports transparent, *publisher-wise* dynamic state repartitioning without client disconnection and with minimal notification delivery interruption for subscribers.

As shown in the evaluation section, E-SilboPS can be successfully used as communication mechanism between IoT devices and any kind of application interested in the data generated by them that implements the proposed general architecture. Moreover, thanks to its elastically-scalable nature, it can sustain the large amount of data that today’s IoT services can generate.

Unlike other proposals such as [16], which focuses on fault tolerance and thus requires more space to store the redundant state and also a more complex algorithm for execution by the *Access Point* layer, or [17], which is really a reimplementation of an application-specific version of the VM migration capability offered by today’s hypervisors, we described the full algorithm that we validated with qualitative and quantitative measurements. This demonstrated that dynamic state repartitioning is not only possible but also achievable even from the performance point of view.

Finally, note that the reported research is being developed and applied as part of the 4CaaSt, FIWARE and FICORE EU FP7 projects, where Publish/Subscribe is offered as both a value-added service to hosted applications and a key internal platform asset.
